# Cold snare unmasking as a one-stop-shop procedure for an unequivocal (tissue) diagnosis of gastric lipomas lacking characteristic findings on standard endoscopy

**DOI:** 10.1097/j.pbj.0000000000000229

**Published:** 2023-10-16

**Authors:** Vincent Zimmer

**Affiliations:** a Marienhausklinik St Josef Kohlhof, Department of Medicine, Neunkirchen, Germany; b Department of Medicine II, Saarland University Medical Center, Saarland University, Homburg, Germany

**Keywords:** lipoma, endoscopy, subepithelial lesion, endoscopic ultrasound, tissue diagnosis

## To the Editor:

**Figure 1.**
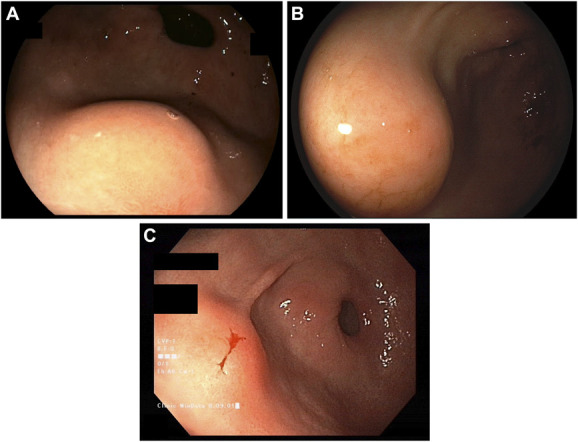
*Patient 1*; (A) depicts an estimated 12-mm SEL in the distal antrum at the 7 o'clock position with prominent luminal bulging. Patient 2; (B) EGD demonstrates a 14-mm lesion in the antrum. Patient 3; (C) provides an endoscopic view of the 10-mm SEL.

**Figure 2.**
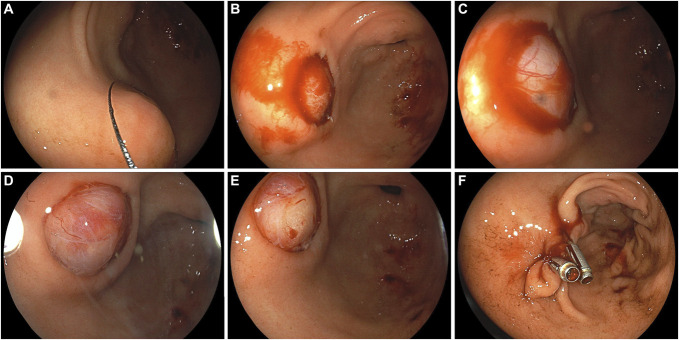
*Patient 2* (male, 72 years); (A) An estimated 14-mm lumen-protruding SEL in the antrum with the cold snare capturing the lesion. (B) Minor oozing after cold snare resection of the overlying mucosa, which is discarded. (C) Close-up view of the surface of the SEL visible under the submucosal plane. (D) After washing, the whole lesion becomes more clearly visible. (E) Further manipulation of the submucosa with a standard forceps results in exposure of a “*naked fat sign*,” signifying an unequivocal lipoma diagnosis, which was further substantiated by histology after biopsies under direct vision (*not shown*). (E) Complete clip closure of the defect.

Gastric subepithelial lesions (SELs) are common in endoscopy practice. Clinical management depends on multiple factors including tissue diagnosis and thus assessment of malignant potential.^1^ While current guideline recommendations prioritize endoscopic ultrasound (EUS) characterization with or without puncture, a one-stop-shop approach to be implemented during index esophagogastroduodenoscopy (EGD) may streamline diagnostics and eliminate the need for ancillary studies and follow-up. Specifically, the recent, albeit mostly of low-to-moderate evidence, authoritative guideline by the European Society of Gastrointestinal Endoscopy suggest EUS characterization and potential puncture for gastrointestinal stromal tumor (GIST)-suspective lesions, at least so if estimated at >20 mm. Alternatively, tissue diagnosis by mucosal incision–assisted biopsy (MIAB), for which small-scale systematic studies are available, is recommended. By contrast, the presence of a lipoma with an unequivocal endoscopic presentation as the most common and harmless type of SEL in the stomach may not warrant specialized diagnostics. While its endoscopic appearance, such as a yellowish aspect and/or a “*naked fat sign*” on standard and/or bite-on-bite biopsy, is appreciable in many cases, an atypical endoscopic presentation is not uncommon and mandates a clear-cut differentiation, in particular, from gastrointestinal stromal tumors (GISTs) at the other spectrum of the malignant potential. However, in equivocal cases, real-time characterization of benign subepithelial lipoma with or without tissue diagnosis may be beneficial in terms of reduced economic (costs, workload of endoscopy units) and, albeit essentially no patient-reported outcome (PRO) data are available to this end, psychological burden on the patient part. Resectional treatment of gastrointestinal lipomas is only indicated in a minor fraction with symptoms of, for example, obstruction and/or hemorrhage.^2^ Therefore, and in extension of a singular case reported elsewhere, pioneering an innovative endoscopic SEL tissue acquisition technique, a more straightforward approach to unmask gastric lipoma, relying on resecting the overlying mucosa by a standard cold snare is presented herein in three consecutive patients with somewhat uncharacteristic standard endoscopy.^3^ Table (Supplemental Content, http://links.lww.com/PBJ/A32) and Fig. 1 present basic patient and SEL characteristics. In addition, Fig. 2 demonstrates the individual steps of the procedure. In brief, the overlying regular mucosa is judiciously resected and discarded, exposing the submucosal plane. Next, submucosal fibers are further pushed aside as appropriate in a blunt fashion and/or grasped by sharp closures of a standard forceps, until a clear-cut “*naked fat sign*” becomes appreciable. Albeit not mandatory by itself, biopsies from visible fat are obtained under direct endoscopic vision and sent for histopathological confirmation of lipoma as tissue diagnosis. As a final step, in this series, the defective lesions underwent complete clip closure to mitigate residual risk of bleeding.

## Conflicts of interest

The authors declare no conflicts of interest.
